# New York Odontological Society

**Published:** 1877-01

**Authors:** 


### ARTICLE II.

### *New York Odontological Society.*

Regular meeting of the society held at the residence of Dr. J. W. Clowes, No. 597 Fifth Avenue, Tuesday evening, June 20th, 1876. President A. L. Northrop in the chair.

The following address was read by Dr. G. N. Winderling, of Milan, Italy:

Gentlemen: Although I am not yet a member of the Odontological Society, I embrace the opportunity offered me to address you in a few words relating to my presence in your country, and to present some considerations on the matter of the exercise of our profession in Italy. As you can be convinced by the official letters I present to you, the Italian Minister of Public Instruction, wishing to establish a special chair of dentistry in the universities, took advantage of my travel, and commissioned me to visit your colleges and institutions having for their object the teaching and improvement of dentistry, and to write at length a complete report of the systems of teaching, and of the programmes of studies and examinations in use in your colleges.

The Minister having heard my views, thought as I did, that he could not have good information and profitable direction but in your country, for America is the only country where dentistry has risen to the degree of a science seriously considered by every person, studied by men of great mind, and practiced by skillful operators. In order to facilitate the means of succeeding, I was accredited from the Italian government to the royal Minister residing in Washington. I was then introduced in Philadelphia, the first city I reached after my arrival, to several colleges by which I was enabled to become acquainted with you. I have visited the colleges of Philadelphia and New York; I know several professors and practitioners, and I take advantage of this meeting to thank them for their hearty and friendly welcome.

I am established as dentist with my brother in Milan, where we succeeded our father, who practiced dentistry in that city during seventeen years. Although living far from America, we have always endeavored to become familiar with your systems and improvements, to say nothing of the different inventions made by my father, for which reason his name is well known to our European colleagues.

In the exercise of our profession we met great obstacles in educating the people, who were almost ignorant of the elementary principals of dental hygiene. We could hardly convince them that it was better to preserve their teeth than to extract them, and it cost years of labor to bring conviction to their minds filled with prejudices. Here, I saw that the people were educated in that matter ; they endure better and without lamentation long operations, having for their object the preservation of the teeth, while in our country we seldom can keep a patient in the chair more than two hours ; he soon becomes tired, excited, and intolerant ; sometimes to satisfy the patient, we are obliged to extract a tooth that could be cured with care and patience. I have seen in your offices many patients, even children, submit to some gold fillings during several hours, helping the operator by their patience, endurance, and will. In a word, your patients have confidence, which with us they lack, because we have not yet special institutions, proving to the patients that the operator has studied and is worthy of their confidence. This want makes the patient doubtful of the capacity of the operator, unless the reputation of the latter is firmly established by years of practice. But now, some institutions based on your example will be created. According to the ideas of the Minister, I suppose that the first chair of dentistry will be established in the faculty of medicine of Pavia, where I graduated. This chair was offered to me, the offering of which I am proud : for if I have no other merits, I have at least this one of having taken the initiative. Knowing the intention of the Minister, my brother and I begun, five years ago, the formation of a museum, designed to illustrate the theory of our profession, representing in wax and natural preparations dental descriptive anatomy and physiology, pathology, surgery, prothesis, and orthopedy. It is composed of one hundred and fifty-four pieces, with some preparations for the microscopial studies, and some minerals, with their products, to demonstrate the ceramic art applied to the manufacture of artificial teeth ; it contains all the preparations necessay for teaching dentistry, that are not found in the numerous museums of general human anatomy.

Now allow me a short criticism: your dental colleges are not provided enough with special preparations of dental anatomy, and I think it would be useful to compose more complete museums to facilitate the teaching to the professors and the learning to the students. My museum is now exhibited at the Centennial Exposition; it will afterwards be employed by the faculty as an illustration to the lessons of dentistry. I will be glad to hear your opinion about it; here I have some samples you can observe now. You will see the entire museum when you will visit the Italian department in the Main Building of the Centennial Exhibition.

Dr. W. H. Atkinson. I think that the paper, in the mere naming of the necessities mentioned, is sufficient to carry conviction to the mind of every person who has tried to teach the advantage of having appropriate objects with which to address himself to the eye of the pupil. Certainly more progress would be made, and more profound knowledge possessed by our body, if that method were practiced. You will observe that every one who has practiced has become more intimate with the principles than those who have tried to get knowledge in almost any other way. We cannot be too thankful to our distinguished guest for bringing us such a suggestion.

Dr. John Allen. At the Centennial I had the pleasure of examining what the doctor terms his museum, and it does present a great variety of anatomical and pathological conditions of the jaws and teeth; and I could not but admire the manner in which the specimens were arranged.

Dr E. A. Bogue. It may have occurred to the minds of all present that the paper of Dr. Wenderling is an important contribution to the history of our calling. We are upon on the eve of having somewhat of a history as pertaining to our own country; and, judging from the workings of the human mind, I think that we, as Americans, are a little apt to think that that means a history of dentistry. It seems to me that this paper is of a character to perhaps open our eyes to what is going on abroad, and to the fact that others, too, are at work. We are told that Italy proposes to establish dental teachings in her university. Looking back to where dentistry was a hundred years ago, I think we should have encouragement and confidence that the time is not far distant when we may indeed take rank as a profession, and when our students will enter those institutions where the most thorough training is to be had, rather than where they may most quickly obtain their diplomas.

Dr. J. Smith Dodge, Jr. I would like to remark upon the suggestion which Dr. Winderling kindly makes upon the lack of museums in colleges in this country. I have had acquaintance with two of them in different capacities, and in neither of them was there anything deserving to be called a museum of dentistry. Why should such a collection necessarily be attached to a college? It would be very useful there; but those of us who are past our college days might derive a great deal of instruction from a collection of the sort named. Why should not this society start a museum of dental objects, making it broad enough to take in everything that the dentist would be especially interested in. I do not doubt that if the gentlemen interested in this society should bring together those various objects of different kinds, now in their private collections, a very respectable beginning for a museum would be made.

Dr. C. A. Woodward presented a boy twelve years of age, for whom, one year ago, had been extracted the right lower sixth year molar; from that time to this a large amount of pus had been constantly discharging through a fistulous opening where the tooth was taken from. The gums appeared quite healthy, no inflammation being apparent. On pressing against the chin on the left side (at times when the face is swollen), pus enough to fill the boy's mouth would be discharged.

Dr. W. H. Atkinson. I am always pained to see this kind of a case in the hands of medical men or even of most dentists, for the reason that they have not given sufficiently close attention to such cases to get at the foundation of the trouble; and they fall into a sort of routine method of management which classifies cases, and then puts them all under one category, for which iodide of potassium, quinine, etc., constitute the treatment; and *that* without any direct reference to the specific objects of the preparation, or to the suiting of the agent to the condition of the body, if it be a tonic. There is so little known about what we mean when we say tonic and stimulant and sedative, and all that, that we seem to be under the necessity of going back to the foundation of things and coming up again in our methods, and have a sort of codification of what is known,—a new and logical arrangement of what we know about the disturbances of function. My idea of a tonic is anything that will supply needed material in the body. Anything that increases or accelerates action in the body, and passes out of the system, is a stimulant; and anything that reduces that action is a sedative. What is the reason that some bodies are cold and others are hot? The reason has never yet been written. I am not sure that I have got the last bottom truth about it myself, but I conceive it to be entirely within the pale of what is called physiological chemistry.

The tumor in this boy's mouth is a case of that low type of functional aberration that we call strumous. The supply of pabulum, out of which the tissues were fed, was not well elaborated; and then the lad has not been required to use his bodily powers to the limit of endurance, as every lad should be in order to get the full endowment of his organization. Hence this accumulation took place, but why it located in the chin I don't know. If the boy can be properly fed, it is self-curative; but it will be a very tedious matter, and may leave indurations that can never be removed.

The treatment I would suggest would be to explore the depths and find whether there is one single sinus from the place where the abscess is discharged, or whether there are little lines that run off, sinuses running hither and thither, and also to ascertain whether it is connected with a necrotic gland, that is, a dead gland. Some one of the glands in the chin may have died ; and if so, that is a point that will need attention, so as to permit the pabulum that is wept out there for the purpose of healing to be built into a new tissue, and not be poured out to be converted into what is called pus. When you have probed and investigated to the bottom and found out where the trouble is, if necessary, make a counter-opening, so that a syringe can wash it out thoroughly.

The constitutional treatment would be iodide of iron, principally ; enough quinine to give his blood the right tone, so that it shall be in proper condition to carry the oxygen to the extremities of the vascular circulation.

I would feed him on the best food I could, and give iodide of iron, much as would digest. You can hardly give too much iodide of iron, because whatever is not appropriated is thrown off; and that, too, is in some sort the case with quinine. The old idea was that quinine was such a terrible thing ; but quinine is expensive and we ought not to waste a particle. The best experiments have shown that no case can ever appropriate more than three grains in one day of twenty-four hours; and for this case I would advise not to exceed three grains in a day of the quinine, and that continued four months or more; find out the food he can most easily digest, the richest you can get; and occassonally give a little potash if the glands show any disturbance.

Dr. W. H. Dwinelle, of New York, read the following paper, entitled " Rapid Operations : "

I think Dr. Lord is entitled to the gratitude of every intelligent member of the profession for his able paper read before this society several months ago, in which he conscientiously directed our attention to what he deemed a serious, if not overwhelming, objection to the burring engine now in use with us. He felt constrained to sound the tocsin of alarm in our camp, and bid us beware lest we might unwitingly be taking an enemy into our hands to steal away our brains and draw down our honored colors from their high standard to trail them in dust and degradation· I certainly honor him for it. It is well for us, in our onward career, occasionally to come to a halt by the wayside, to look backward as well as forward, and, in the light of the present, to note and compare the two extremes, that we may faithfully and healthfully make honest deductions and conclusions, in order to be assured that our career is indeed onward.

I rejoice that there exists this spirit of conservatism in our midst, even though it be in extreme ; so long as it checks and restrains the over-enthusism and dangerous confidence that characterizes many in our profession, who are too ready to adopt and hastily indorse the " NEW " before they have given it the test of time. Our calling should be too valuable to us. The permanent interests of our patients, and the sacred trust committed to our charge should make us cautious, conscientiously to deliberate in deciding upon the mer'ts of all new instrumentalities, that may prove dangerous in our hands.

Although this subject has, since Dr. Lord's initial paper, been discussed somewhat extensively, still, its importance, I think, will justify me in asking your further attention briefly this evening.

In some respects the engine has great advantage over hand-working. The absolute precision and steadiness with which you can accomplish your purpose, especially in much of the excavating department; the right and other angle attachments, and the revolving disks ; the burring, condensing, polishing, burnishing, and finishing of much of our gold stopping work, place the engine, as an aid in these directions, pre-eminently above all other methods hitherto known in our art, and make it, in my opinion, an indispensible auxiliary to every conscientious, capable, and progressive dentist, who studies alike the interests of his patients and himself.

Lest I may do injustice to any one by an assertion seemingly so bold as the one just uttered, I will qualify it by saying that, whereas the engine is, as a rule, most dangerous in the hands of the young and inexperienced, yet there are "children of older growths" who, while comparatively successful with old methods, are, from the force of long habits in prescribed paths, from a prejudice against the new, and from a certain constitutional inaptitude, honestly incapable of doing justice, with the engine, to themselves or their patients.

This, however, proves nothing against the engine, which, in the hands of the skillful, the experienced, and the appreciative, has accomplished so much,—it simply proves that these individual practitioners have not been successful, and that, if we are to accept their testimony, they cannot be.

"Our art's unsparing rules
Give us the handling of her sharpest tools."

We can use them for weal or woe. In the hands of intelligence and skill, they may be guided to the alleviation of pain and to permanent restoration ; in those of ignorance and inexperience, to unnecessary suffering, to destruction and disappointment. In neither case should the instrument be accredited or condemned for the result, but the inspiration, the intelligence, and skill—or their negation—that stood behind it.

In my opinion, the engine should always be used as an auxiliary ; the old method and the old instruments should always be used in connection with it. The engine never exclusively, nor to the abandonment of the standard methods and instruments established by long experience, and by the verdict of our best, in the past and the present. In many operations, and details of operations, it is vastly superior to the ordinary hand instruments, but in many others it is vastly inferior, and is utterly incapable of being substituted for them.

The highest expression of an instrument which shall be a medium of manipulation to an intelligent end is one that is so balanced as to weight, as to length, as to quality, as to sharpness or acuteness, as to delicacy and true relation to the inevitable and fixed laws of form, that it shall seemingly have such an appreciation of means to ends, and shall be so weighted,—and resting, not grasped with arbitrary over-firmness,—in the hand of the operator, that he shall be enabled to project himself, his perceptions, and his intelligence through it, and beyond it, to the end to be accomplished.

Such an instrument can only, and must ever be, a hand instrument! With and through such an instrument you become endowed with such a fine and delicate sense of quality that, even with your eyes shut, you are enabled to judge of and feel the varying textures of the entire interior of the field of your labor, whether they be soft or hard decay, dentine or enamel. There is a subtle link of sympathy and appreciation—I had almost said sentiment—between you.

With the painter, his brush is this medium of communication and expression of his interior inspirations. With the sculptor, it is his modeling tool and his chisel. With the musician, his harp, his viol, or his organ.

This exaltation must ever be in proportion as the barriers, interferences, and impediments between the operator and the operation under performance are removed.

You have all of you read learned discussions upon the proper length that should be given the handle to render the hammer effective.

Your barber, if he be an artist, and many of them are, will tell you that, to get the finest and nicest practical sense of touch of the razor, while in use, you should have it so nicely balanced in your fingers, and should hold it with such a delicate grasp, that should one lightly hit your elbow, while using it, it would drop from your hand.

A skillful and sensitive surgeon will feel the different textures of cuticle, flesh, fiber, or cartilage as he passes through them with his scalpel.

For a large class of delicate operations the engine should never be used. If you attempt to do so, you will find yourself losing that independent sense of guidance; you will have to overcome an arbitrary deadness, an inert power of resistance, fixed in opposition to yourself. Your fine sense of touch is crippled, confined, and hampered; you become subjective instead of positive,—the subtle link of sympathy, of touch, of feeling, of appreciation, is broken; all sense of quality is gone from you, and perhaps you will learn the unwilling lesson that none of these can ever be transmitted through the very useful and practical burring engine.

We cannot but concede the fact that it is a serious objection to the engine, in that it presents such a formidable array to our sensitive patients, frightfully suggestive of the gillotine, and of being ground, tortured, and lacerated by machinery.

The jar and vibration which is transmitted by it through the whole physical system is more than some natures can bear. The heating of the teeth by friction, unless great care is used, and the fact that, with the best of them, the motion is so rapid that it defeats itself, so that sometimes a few rotations of a hand-bur, or drill, are more effective than a thousand revolutions of the engine.

It scuds over the surface, and does not lay hold on the dentine to be excavated. The paddle-wheel of a steamboat may be made to rotate so rapidly that it will not lay hold on the water, and the boat will come to a stand-still, the water winding around it like a skein of silk.

Again, some points will cut so much more rapidly than others that, unless great care is used, you first make the discovery by finding you have exposed a nerve, transfixed a tooth, or undermined a wall that might have served you for a sure foundation.

These enumerated are some of the objections to the engine, many of which may be overcome; and, although it can never approach the character of, or be a substitute for, the hand instrument in a large class of delicate operations, it is, as an auxiliary, one of the most valuable contributions to our profession that has ever been made, and will always command our gratitude and appreciation.—*Dental Cosmos.*

[TO BE CONTINUED.]

